# Hearing Loss in Patients with Systemic Lupus Erythematosus

**DOI:** 10.5539/gjhs.v5n5p102

**Published:** 2013-06-15

**Authors:** Mahnaz Abbasi, Zohreh Yazdi, Amir Mohammad Kazemifar, Zahra Zarin Bakhsh

**Affiliations:** 1 Rheumatologist, Metabolic Disease Research Center, Qazvin University of Medical Sciences, Qazvin, Iran; 2 Occupational Medicine Specialist, Metabolic Disease Research Center, Qazvin University of Medical Sciences, Qazvin, Iran; 3 Clinical Toxicologist, Qazvin University of Medical Sciences, Qazvin, Iran; 4 General Practitioner, Qazvin, Iran

**Keywords:** systemic lupus erythematosus, sensorineural hearing loss, auditory threshold

## Abstract

**Objectives:**

Systemic lupus erythematosus has its unique complications which warrant careful examination and assessment during follow/up visits of patients. The present study was conducted to evaluate prevalence of hearing loss in patients with SLE.

**Materials & Methods:**

At present a case- control study has been performed on 45 patients with SLE in a clinic of a teaching university hospital, Qazvin city, Iran. The patients were examined and evaluated for auditory and hearing problems as well as parameters related to their disease severity and progression. The control group was selected from the same clinic.

**Results:**

Five patients (11.1%) complained from hearing loss, 4 patients (8.9%) complained from otorrhea, 3 patients (6.7%) had tinnitus in research group, moreover twelve patients (26.7%) in case group and 4 patients (8.9%) in control group had sensorineural hearing loss. The difference was found to be statistically significant. No statistical significant relationship was found between severity, age of onset, and duration of the disease, and the lab tests of the patients with hearing loss.

**Conclusion:**

The present study implies that patients with systemic lupus erythematosus may develop sensorineural hearing loss during their course of the disease. It is recommended that audiology examination and/or audiometry become a part of routine follow/up studies of the patients.

## 1. Introduction

Systemic lupus erythematosus (SLE) is an autoimmune disorder with multi-organ involvement. The main mechanisms of the disease are immune complex formation and production of auto-antibody (Karatas et al., 2007). Recently, it has been suggested that immunologic processes may involve inner ear (Ruckenstein, 2004; Mathews et al., 2003). It is true for SLE too. Kastaniousdakis et al. (2002) and Sperling et al. (1998) have reported that patients with SLE may complain from auditory symptoms. Furthermore, various studies proposed a number of mechanisms such as vasculitis processes or free radicals formation in steria vessels of Choclea (similar to animal models of lupus), autoimmunity due to vasculitis, early presbycusis, drugs toxicity, micro-infarctions in capillaries and arterioles of temporal bone, thrombosis in the vessels of ear, and anti-phospholipid syndrome to deduce mechanism(s) of auditory involvement in SLE (Rukenstein et al., 1999; Andonopoulos et al., 1995; Bortoli et al., 2007; Caladerli et al., 1986; Hisashi et al., 1993).

Different studies have reported diverse rates for prevalence of auditory disturbance in SLE. Auditory disturbance has been reported in 8-66 percent of patients with SLE (Karatas et al., 2007; Kastanioudakis et al., 2002; Sperling et al., 1998; Andonopoulos et al., 1995; Skrzypezak et al., 2006; Gomides et al., 2007; Bowman et al., 1986; Roverano et al., 2006). In a study, hearing threshold had been declined in all frequencies except 2000 to 4000 Hertz in patients with SLE (Maciaszczyk et al., 2011), while decline in hearing threshold has been more prominent at 2000 to 4000 Hertz; like an early presbycusis in another study (Quick et al., 1973).

Because of inconsistency in the reported related studies, the present study was conducted to evaluate auditory disturbance in patients with SLE and its correlation with severity and duration of the disease.

## 2. Methods

A case-control study was performed on patients who fulfilled at least four international criteria established by the American Rheumatism Association, revised criteria for SLE (Tan et al., 1982). They were selected from patients referred to rheumatology outpatient clinic of a university teaching hospital in Qazvin city, Iran. Exclusion criteria comprised; (a) past history of ear infection or discharge, (b) head trauma, (c) hearing loss before onset of SLE, (d) head and neck congenital malformation, (e) family history of early presbycusis, (f) working or living in noisy places and, finally, (g) the use of autotoxic drugs; like high dose ASA, streptomycin, or gentamicin. In total, 45 patients with SLE were enrolled in the study, after the exclusion of 7 patients (who had one or more exclusion criteria).

The control group comprised 45 individuals that were selected from healthy people who had referred to the same clinic for pre-employment medical examination, if they had not exclusion criteria mentioned in above paragraph. Their age and sex had been matched to the case group.

Demographic characteristic of the patients and the presence of possible auditory related complaints such as tinnitus, sensation of fullness in air, and otorrhea were questioned and collected in checklists. All of the patients were examined by a rheumatologist and the required lab study for assessment of disease severity and activity including Cell Blood count (CBC), Urine Analysis (U/A), Blood Urea Nitrogen (BUN), Creatinine (Cr), Anti-Nuclear Antibody (ANA), Anti double stranded-DNA (Anti-Ds DNA), complement components (C3 and C4) were requested. The disease severity index was determined for the patients according to the standard criteria with systemic lupus disease activity index (SLEDAI) (Skrzypezak et al., 2006; Sperling et al., 1998). They were also evaluated by an otorhinolaryngologist for external ear examination, audioscopy, and audiometry tests, such as PTA (pure tone audiometry), SDS (speech discrimination test), and SRT (speech reception threshold). Tympanometry was performed at 250, 500, 1000, 2000, 4000, and 8000 Hertz.

The collected data was analyzed using SPSS software version 16.0. Chi-square and Fischer exact test were used for comparison of values between the experimental and control groups.

The study has been approved by local ethical committee of Qazvin University of medical sciences. All of the patients had been provided informed consent for contribution to the study.

## 3. Results

The mean age of the studied individuals was 34.9±7.9, and 34.2±7.2 years in case and control groups respectively. Female/male ratio was 13.92 and 8.00 in case and control groups respectively. Differences between groups were not statistically significant.

The mean age of onset of the disease was 30.5±7.4 years. Mean duration of the disease was 4.4±3.3 years (1- 12 years).

In the case group, 5 patients (11.1%) complained from hearing loss, 4 patients (8.9%) complained from otorrhea, 3 patients (6.7%) had tinnitus, and 1 reported sensation of fullness in ear. No similar complaints were reported in control group. Results of the hearing assessment of the groups are shown in Tables 1 and 2.

**Table 1:** Air conduction hearing thresholds in experimental and control group (mean ± SD, the range in parenthesis)

**Frequency (Hertz)**	**Right ear**			**Left ear**		

	Control group	Case group	p-value	Control group	Case group	p-value
250	4.9 (10-30)±17.8	2.8 (10-20)±13.9	0.045	10.3 (5-45)±18.3	4.1 (5-35)±15	<0.001
500	4.9 (10-30)±19.5	1.8 (10-20)±14.2	0.002	9.3 (10-45)±20.3	4.1 (0-20)±15.5	<0.001
1000	4.3 (5-35)±28.8	1.9 (10-15)±14.1	0.759	8.1 (5-55)±14.8	2.8 (0-20)±15.9	0.298
2000	6.8 (0-30)±11.4	2.3 (0-10)±8.5	0.204	9.7 (5-50)±20	2.5 (5-15)±10.1	<0.001
4000	6.3 (5-25)±12.2	3.7 (5-15)±9.2	0.012	9.4 (5-45)±12.7	4.5 (0-15)±8.7	0.007
8000	10.3 (5-55)±17.2	3.7 (5-35)±10.7	0.001	14 (0-55)±17.5	3.5 (5-15)±10	<0.001
Mean	4.5±15.7	1.6±11.8	0.031	9.3±16	4.9±12.6	0.03

**Table 2:** Bone conduction hearing thresholds in research and control group (mean± SD, the range is in parenthesis)

**Frequency (Hertz)**	**Right ear**			**Left ear**		

	Case group	Control group	p-value	Case group	Control group	p-value
250	4.5 (5-20)±12	0 (10-15)±10	0.003	4.7 (5-25)± 11.9	2.2 (5-15)±9.9	0.012
500	4.2 (5-25)±13.8	1.3 (5-15)±10.3	<0.001	5.1 (5-30)±13.7	3.3 (5-15)±11.5	0.024
1000	3.7 (5-25)±10.3	2.1 (0-10)±9.2	0.087	4.8 (0-15)±8.6	2.8 (5-15)±8.2	0.595
2000	6.5 (5-25)±6.6	1.6 (0-10)±5.5	0.329	8.2 (5-35)±6.3	1.6 (0-15)±4.7	0.133
4000	5.7 (0-20)±7.1	3.1 (0-15)±5.1	0.041	5.1 (5-15)±6.7	3.4 (0-15)±4.9	0.082
Mean	3.1±10	0.8±8	0.001	4.2±9.4	2.1±7.8	0.021

Twelve patients (26.7%) in the case group and 4 patients (8.9%) in the control group had sensorineural hearing loss. The difference was statistically significant (p-value= 0.049). Relative risk for sensorineural hearing loss was 3.7 (95% confidence interval, 2.3-4.9) in patients with SLE.

In case group, active dermal, joint, and renal manifestations were found in 6 (13.3%), 7 (15.6%), and 2 (4.4%) of the patients respectively. No relationship was found between activity of the disease and hearing loss. The patients were using prednisolone, hydroxychloroquine, and methotrexate for their disease. No relationship was confirmed between types of the drug used by the patients and the audiometry tests.

Result of the various lab tests of the patients which categorized by result of audiometry are evident in table 3. No relationship was found between them.

**Table 3:** Relationship between lab data of the patients in case group and their audiometry

Lab data	Result	Number	Frequency (percent) of hearing loss	P Fisher's
Anti-ds DNA	High	12	3 (26.7%)	0.467
	Normal	23	9 (73.3%)	
ANA	Positive	20	6 (44.4%)	0.730
	Negative	25	6 (55.6%)	
C_3_	Low	10	3 (22.2%)	0.687
	Normal	35	9 (77.8%)	
C_4_	Low	9	4 (20%)
	Normal	36	8 (80%)	1

The disease severity index (SLEDAI) was determined for the patients. No relationship was found between the index and hearing loss. The details are presented in table 4 below

**Table 4:** Relationship between the Disease severity index (SLEDAI) and hearing loss in the patients of case group

Hearing loss	SLEDAI 0-5	SLEDAI 6-10	SLEDAI >10	P fishers
Yes	10	2	0	0.169
No	32	1	0	

Furthermore, no relationships were found between duration and age of onset of the disease, and hearing threshold.

SDS test of 4 patients (8.9%) in the research group was abnormal. No abnormality was found in SDS test of the control group, however the difference was not statistically significant (P- Fischer= 0.117).

SRT test of both groups were within normal range. The mean score of SRT test was 12.6 and 16.8 in the case and control group respectively. The difference was not statistically significant (p-value= 0.119).

## 4. Discussion

Results of present study confirmed that patients with SLE have lower hearing threshold compared to normal individuals. Their hearing threshold was roughly 2-3.5 dB lower than the matched control group.

Moreover 26.7% of the research sample had high frequency sensorineural hearing loss (SNHL) which was much higher than control group. This is similar to the study of Maciaszczy and colleagues (2011) who have reported high frequency SNHL in 28.6% of patients with SLE. Roverano et al. (2006) has also found high frequency SNHL in 66% of patients in his study on 31 patients with SLE. Nevertheless, it is believed that hearing loss will be typical for auto-immunity, if it is bilateral, asymmetrical, and fluctuating, and involves medium frequencies initially (Ruckenstein, 2004). Karabulat and colleagues (2010) has also reported lower frequencies hearing loss (i.e. typical of auto-immune hearing loss) in patients with SLE.

It is generally expected that auto-immune hearing loss may occur unilaterally. Karatas et al. (2007) has reported SNHL in 21% of patients with SLE, which was unilateral in half of the cases. Furthermore, Sperling et al. (1998) has reported unilateral and bilateral hearing loss in 15% and 17% of 84 patients with SLE. However, hearing loss was bilateral in all patients examined in current study. It is comparable to the studies of Roverano et al. (2006) and Maciaszczy et al. (2011); though the reported frequency of bilateral SNHL has been much lower compared to study of Roverano.

No relationship was found between aural related symptoms; like tinnitus, sensation of fullness in ear and otorrhea and SNHL in current study. It implies that patients with SLE may develop SNHL without alarming symptoms; which might be noticed by their physicians. Sperling et al. (1998) had been found relationship between auditory symptoms and SNHL in his study. Relative risk for bilateral SNHL was calculated 3.7 in patients with SLE compared to normal individuals in the present study. It is reported to be as high as 20 in the study of Roverano (2006). He had examined 30 lupus patients and reported asymptomatic SNHL in 20 of 30 (66%) of them, so the relative risk of patients to experience SNHL was 20 in comparison to control group.

There was no association between duration of the disease and hearing threshold in the present study. It is contrary to study of Maciaszczyk (2011) which had reported significant positive correlation between air conduction hearing loss and duration of SLE. Also hearing loss was not associated with SLEDAI score in the current study. Roverano (2006) and Maciaszczy (2011) have found similar results. In addition, serum concentration of C_3_, C_4_, creatinine, and ESR were not correlated with hearing loss or hearing threshold in the studied patients, whereas Sperling (1998) has suggested that rise in creatinine level and decline in C_3_ are correlated with aural symptoms.

There were few previous studies that had suggested relationship between SNHL and SLE. However similar study had not been performed among Asian population, yet. Moreover, various mechanisms have been proposed for higher prevalence of SNHL among SLE patients (Rukenstein et al., 1999; Andonopoulos et al., 1995; Bortoli et al., 2007; Caladerli et al., 1986; Hisashi et al., 1993). Results of current study may imply that SNHL is not directly related to the disease process; since is not related to the severity of the disease. Other potential mechanism should be taken into concern. More detailed study should be designed about the matter to elucidate the mechanism. If audiology examination of the patients is conducted as soon as the diagnosis is made, more conclusive results about the mechanism and pathophysiology of SNHL may attain.

There were some limitations in present study. Small sample size is one of them. Moreover, we had not any information about the time of start of SNHL. None of the studied patients had undergone audiology examination before the study. This might help us to reach to more decisive conclusion.

## 5. Conclusion

In summary, the results of present study suggest that patients with SLE may develop SNHL as a consequence of their disease. It is recommended that E.N.T examination and/or audiometry become a part of routine follow/up visits of the patients.
